# Salvianolic Acid A Activates Nrf2-Related Signaling Pathways to Inhibit Ferroptosis to Improve Ischemic Stroke

**DOI:** 10.3390/molecules30153266

**Published:** 2025-08-04

**Authors:** Yu-Fu Shang, Wan-Di Feng, Dong-Ni Liu, Wen-Fang Zhang, Shuang Xu, Dan-Hong Feng, Guan-Hua Du, Yue-Hua Wang

**Affiliations:** Beijing Key Laboratory of Innovative Drug Discovery and Polymorphic Druggability Research for Cerebrovascular Diseases, Institute of Materia Medica, Chinese Academy of Medical Sciences & Peking Union Medical College, Beijing 100050, China; shangyufu1998@163.com (Y.-F.S.); fwandi@imm.ac.cn (W.-D.F.); liudongni@imm.ac.cn (D.-N.L.); zhangwenfang@imm.ac.cn (W.-F.Z.); xushuang@imm.ac.cn (S.X.); fengdanhong@imm.ac.cn (D.-H.F.); dugh@imm.ac.cn (G.-H.D.)

**Keywords:** ischemic stroke, ferroptosis, salvianolic acid A, nuclear factor erythroid-derived 2-related factor 2, lipid peroxidation

## Abstract

Ischemic stroke is a serious disease that frequently occurs in the elderly and is characterized by a complex pathophysiology and a limited number of effective therapeutic agents. Salvianolic acid A (SAL-A) is a natural product derived from the rhizome of *Salvia miltiorrhiza*, which possesses diverse pharmacological activities. This study aims to investigate the effect and mechanisms of SAL-A in inhibiting ferroptosis to improve ischemic stroke. Brain injury, oxidative stress and ferroptosis-related analysis were performed to evaluate the effect of SAL-A on ischemic stroke in photochemical induction of stroke (PTS) in mice. Lipid peroxidation levels, antioxidant protein levels, tissue iron content, nuclear factor erythroid 2-related factor 2 (Nrf2), and mitochondrial morphology changes were detected to explore its mechanism. SAL-A significantly attenuated brain injury, reduced malondialdehyde (MDA) and long-chain acyl-CoA synthase 4 (ACSL4) levels. In addition, SAL-A also amplified the antioxidative properties of glutathione (GSH) when under glutathione peroxidase 4 (GPX4), and the reduction in ferrous ion levels. In vitro, brain microvascular endothelial cells (b.End.3) exposed to oxygen-glucose deprivation/reoxygenation (OGD/R) were used to investigate whether the anti-stroke mechanism of SAL-A is related to Nrf2. Following OGD/R, ML385 (Nrf2 inhibitor) prevents SAL-A from inhibiting oxidative stress, ferroptosis, and mitochondrial dysfunction in b.End.3 cells. In conclusion, SAL-A inhibits ferroptosis to ameliorate ischemic brain injury, and this effect is mediated through Nrf2.

## 1. Introduction

Ischemic stroke (IS), a brain condition resulting from localized blockage of blood vessels, constitutes about 87% of all stroke incidents [1]. Its main symptoms include impaired motor coordination and cognitive dysfunction, with severe cases involving seizures or fatalities [2]. Following IS, thrombotic occlusion hinders the supply of nutrients essential for cell survival, ultimately leading to brain cell death [3]. Photochemical induction of stroke (PTS) is a method used in IS research. It works by exposing photosensitive dyes to certain wavelengths of light, which generate free radicals that damage endothelial cells and trigger the endogenous coagulation pathway, resulting in thrombus formation [4,5]. Besides, PTS offers advantages such as minimal trauma, good stability, and similarity to the process of thrombus formation in the human brain [6].

Ferroptosis was first discovered by the laboratory of Brent Stockwell in 2012. It is named after the excessive accumulation of ferrous ions (Fe^2+^) within cells [7]. The excess intracellular free Fe^2+^ interacts with hydrogen peroxide, triggering the Fenton reaction [8]. This process will further oxidize cellular lipids, deplete antioxidant molecules such as glutathione (GSH) and reduced form of nicotinamide adenine dinucleotide phosphate (NADPH), and induce mitochondrial dysfunction [9]. Additionally, cells undergoing ferroptosis exhibit morphological deterioration of mitochondria [10]. Although the molecular mechanisms underlying ferroptosis after IS are not investigated clearly, it is closely associated with a series of oxidative stress-induced molecular cascade reactions [11]. A considerable quantity of polyunsaturated fatty acids (PUFAs) is found in the human brain, and the generation of reactive oxygen species (ROS) from oxidative stress after IS causes PUFA peroxidation and further exacerbation of ferroptosis [12]. Oxidative stress triggered by IS can result in the deterioration of the blood-brain barrier, make iron deposition easier and potentially trigger ferroptosis [13].

Nuclear factor erythroid 2-related factor 2 (Nrf2) is a critical regulatory protein that modulates the cellular defense mechanisms against ROS, with seven conserved Neh structural domains that regulate various target proteins against oxygen radical attack [14]. Normally, kelch-like ech-associated protein 1 (Keap1) acts as a negative regulator of Nrf2 in the extra-nucleus [15]. Nrf2 then targets nucleus antioxidant response elements (AREs) to trigger the up-regulation of various target proteins such as superoxide dismutase (SOD) and NADPH quinone oxidoreductase 1 (NQO1) [16]. The association of Nrf2 with ferroptosis has been proven in several studies. Many target proteins of Nrf2 are effective in inhibiting abnormalities of lipid metabolism. For instance, peroxisome proliferators-activated receptors γ (PPARγ) is involved in the decomposition of lipid peroxides [17]. A series of GSH-related enzymes such as solute carrier family 7 member 11 (SLC7A11/xCT) and glutathione peroxidase 4 (GPX4) can also be influenced by Nrf2 [18]. Moreover, Nrf2 is involved in maintaining iron homeostasis, as it affects the ferritin heavy chain (FTH) and transferrin receptor 1 (TFR1) [19].

Salvianolic acid family is a group of phenolic acid compounds from the natural medicinal plant *Salvia miltiorrhiza* [20]. Its co-existence with diterpenoids in the roots of *Salvia miltiorrhiza*. Salvianolic acid family contains caffeic acid as a structural unit, which leads to different multimers. The main multimers include dimers (rosmarinic acid), trimers (salvianolic acid A, SAL-A), tetramers (salvianolic acid B), and others [21]. Numerous studies have reported that SAL-A had neuroprotective effects against IS [22,23]. SAL-A may slow the progression of ferroptosis. This study was conducted to investigate whether the mechanism of SAL-A against IS was related to the activation of Nrf2 and its downstream iron metabolism-related cascade proteins.

## 2. Results

### 2.1. Salvianolic Acid A Reduced Ischemic Brain Injury in PTS Mice

**Figure 1:**
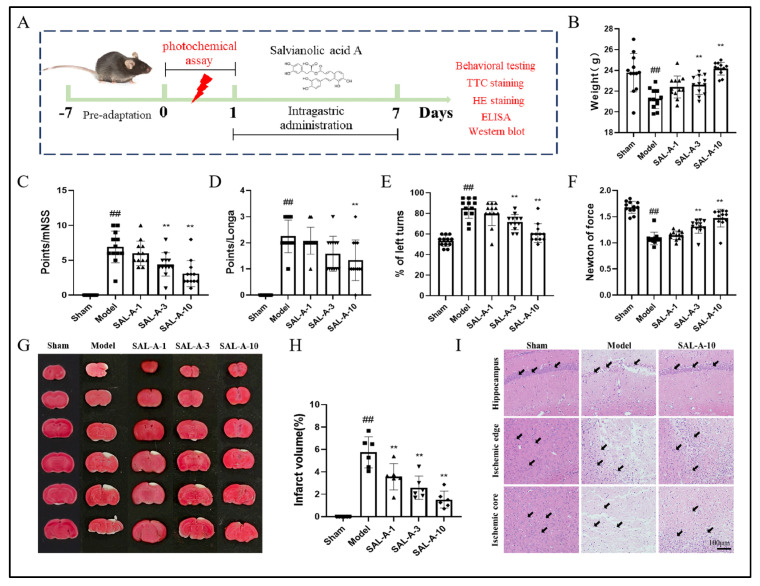
Salvianolic acid A reduced ischemic brain injury in PTS mice. (**A**) Experimental design process. Body weight (**B**), mNSS scores (**C**), Longa scores (**D**), Left turn rate (**E**), Grip strength (**F**) of PTS mice on day 7 (*n* = 12). Representative images of TTC staining (**G**) and quantitative analysis of cerebral infarct volume (**H**) of PTS mice on day 7 (*n* = 6). (**I**) Representative images of HE staining in CA1 field of hippocampus, ischemic edge and ischemic core of the brains of PTS mice (*n* = 4). Values are expressed as Mean ± SEM. ^#^ *p* < 0.05, ^##^ *p* < 0.01 vs. Sham group. * *p* < 0.05, ** *p* < 0.01 vs. Model group.

To investigate the protective effects of SAL-A on ischemic brain injury in PTS mice, different doses of SAL-A were administered for 7 days after the successful establishment of ischemic stroke (Figure 1A). The results suggested that SAL-A dose-dependently improved the body weight recovery rate in PTS mice (Figure 1B). SAL-A also ameliorated neurobehavioral deficits, increased mNSS and Longa scores, improved the turning defect and enhanced grip strength in PTS mice (Figure 1C–F). Additionally, TTC staining results demonstrated that SAL-A dose-dependently reduced the infarct volume in PTS mice, with a dosage of 10 mg/kg showing the most effective neuroprotective impact (Figure 1G–H). To further observe the protective effects of SAL-A on brain tissue cells and neurons, HE staining was performed on PTS mouse brain tissue. HE staining revealed abnormal cell morphology, condensed nucleoli, and disorganized arrangement in the CA1 field of the hippocampus, ischemic edge and ischemic core regions of PTS mice, whereas SAL-A significantly decreased the cell damage caused by PTS (Figure 1I).

### 2.2. Salvianolic Acid A Exerted Antioxidative Stress Effect in PTS Mice

**Figure 2:**
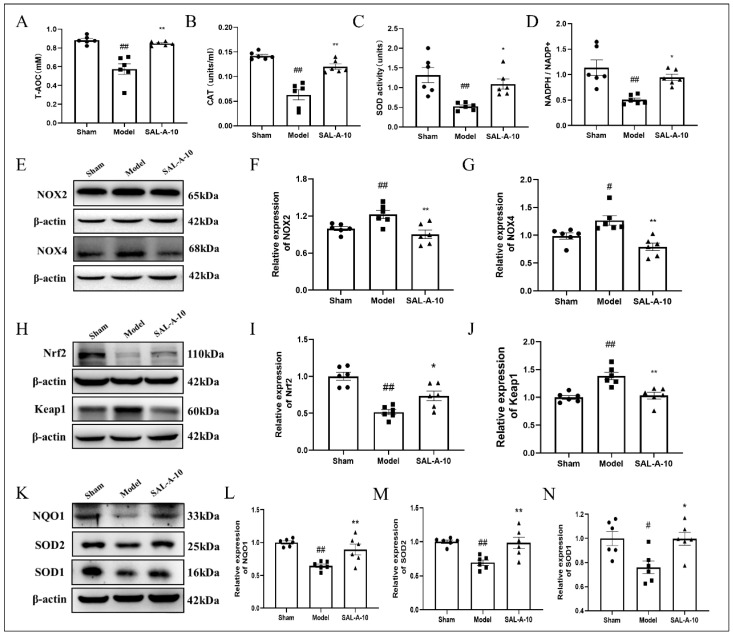
Salvianolic acid A exerted antioxidative stress effect in PTS mice. The levels of T-AOC (**A**), CAT (**B**), SOD (**C**), NADPH/NADP^+^ (**D**) in the brains of PTS mice (*n* = 6). Representative images of Western blot (**E**,**H**,**K**) and statistical analysis showing the NOX2 (**F**), NOX4 (**G**), Nrf2 (**I**), Keap1 (**J**), NQO1 (**L**), SOD1 (**M**), SOD2 (**N**) in the brains of PTS mice (*n* = 6). Values are expressed as Mean ± SEM. ^#^ *p* < 0.05, ^##^ *p* < 0.01 vs. Sham group. * *p* < 0.05, ** *p* < 0.01 vs. Model group.

The study examined SAL-A’s defense against oxidative stress caused by ischemic stroke, and the total antioxidant capacity of PTS mice was assessed. The results showed that SAL-A increased the total antioxidant capacity (T-AOC) in the brains of PTS mice (Figure 2A). Furthermore, SAL-A enhanced the activities of catalase (CAT) and SOD, which are the two major antioxidant enzymes (Figure 2B–C). NADPH is an important electron donor for the clearance of peroxides by GSH in the cell, and SAL-A increased the levels of NADPH/NADP^+^ in the brains of PTS mice (Figure 2D). Additionally, SAL-A reduced the expression of NADPH oxidases 2 (NOX2) and NOX4, thereby interfering with their function in generating ROS production (Figure 2E–G). Nrf2, the main antioxidant stress transcription factor in the cell, revealed a marked reduction in the expression levels within the brains of PTS mice. SAL-A increased the levels of Nrf2 and decreased the levels of Keap1 (Figure 2H–J). Additionally, SAL-A increased the levels of Nrf2 downstream antioxidant proteins, including NQO1, SOD1, and SOD2 (Figure 2K–N).

### 2.3. Salvianolic Acid A Protected from Ferroptosis by Inhibiting Lipid Peroxidation and Iron Overload in PTS Mice

**Figure 3:**
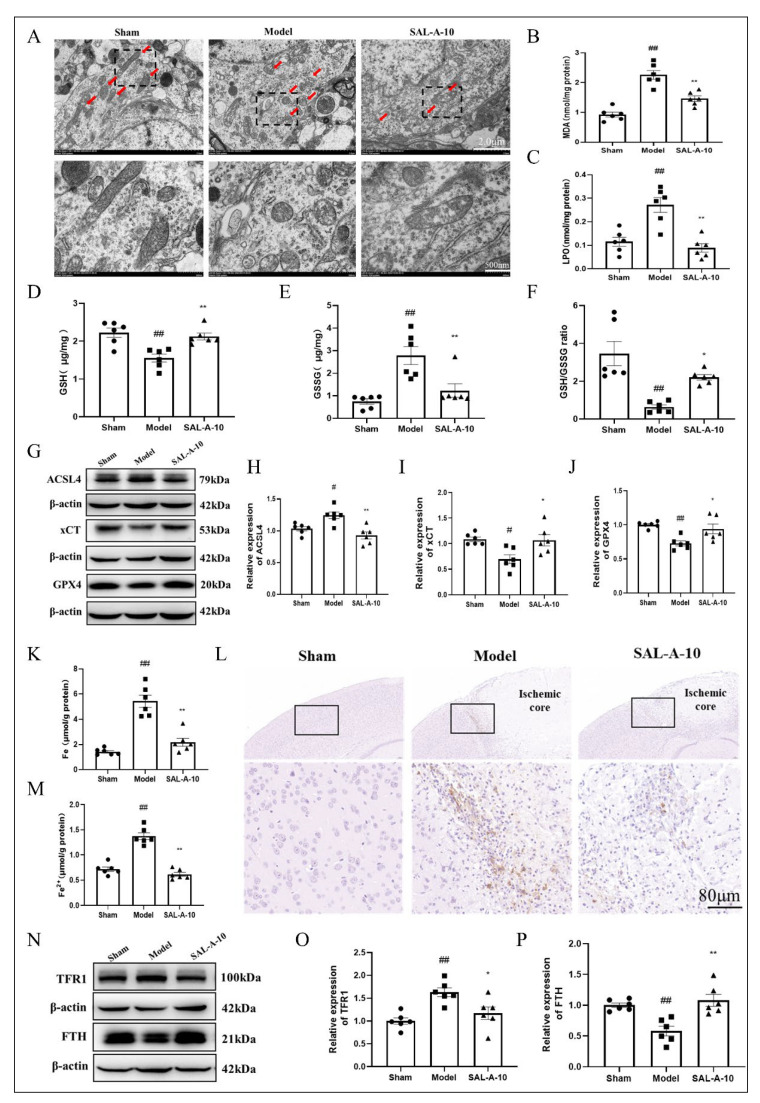
Salvianolic acid A protected from ferroptosis by inhibiting lipid peroxidation and iron overload in PTS mice. (**A**) Representative images of TEM at the infarct border zone in the brains of PTS mice (*n* = 4). The levels of LPO (**B**), MDA (**C**), GSH (**D**), GSSG (**E**), GSH/GSSG (**F**) in the brains of PTS mice (*n* = 6). Representative images of Western blot (**G**) and statistical analysis showing the ACSL4 (**H**), xCT (**I**), GPX4 (**J**) in the cerebral tissue of mice with PTS (*n* = 6). The levels of Fe (**K**), Fe^2+^ (**M**) in the cerebral tissue of mice with PTS (*n* = 6). (**L**) Representative images of Prussian blue staining (DAB enhanced) in the brains of PTS mice (*n* = 4). Representative images of Western blot (**N**) and statistical analysis showing the TFR1 (**O**), FTH (**P**) in the cerebral tissue of mice with PTS (*n* = 6). Values are expressed as Mean ± SEM. ^#^ *p* < 0.05, ^##^ *p* < 0.01 vs. Sham group. * *p* < 0.05, ** *p* < 0.01 vs. Model group.

Various factors that induce ferroptosis, including lipid peroxidation that generates ROS and excess free iron, can disrupt mitochondrial metabolism, leading to alterations such as mitochondrial shrinkage, rounding, and a decrease in cristae. Transmission Electron Microscopy (TEM) results indicate abnormal mitochondrial morphological changes in the infarct border zone of brain tissue in PTS mice. However, the administration of SAL-A has been shown to help maintain normal mitochondrial morphology (Figure 3A). Lipid peroxidation and iron overload are the main regulatory pathways of ferroptosis. Findings indicated that SAL-A lowered the amounts of lipid peroxidation substances, such as lipid peroxidation (LPO) and malondialdehyde (MDA), in the brains of PTS mice (Figure 3B,C). GSH is an important antioxidant that helps cells eliminate LPO. SAL-A increased the concentration of GSH and the reduction of GSSG levels in the brains of PTS mice (Figure 3D,F). ACSL4 serves as a critical factor in the peroxidation of PUFA, and SAL-A can reduce ACSL4 levels in PTS mice. GSH synthesis requires cysteine input through xCT, and GPX4 is essential for the clearance of peroxides by GSH. Our results demonstrate that SAL-A exhibits a beneficial promotive effect on xCT and GPX4 in PTS mice, indicating that ferroptosis is suppressed. (Figure 3G–J). Iron overload is a critical trigger for ferroptosis and results showed that SAL-A decreased the concentrations of Fe and Fe^2+^ in the brains of PTS mice (Figure 3K,M). Prussian blue staining (DAB enhanced) also demonstrated a reduction of brown iron deposits (Figure 3L). TFR1 is responsible for iron uptake, while FTH can regulate iron storage in cells. SAL-A decreased TFR1 and increased FTH levels, thereby reducing iron overload (Figure 3N–P).

### 2.4. Salvianolic Acid A Attenuates OGD/R-Induced Oxidative Stress in b.End.3 Cells via the Nrf2 Signaling Pathway

**Figure 4:**
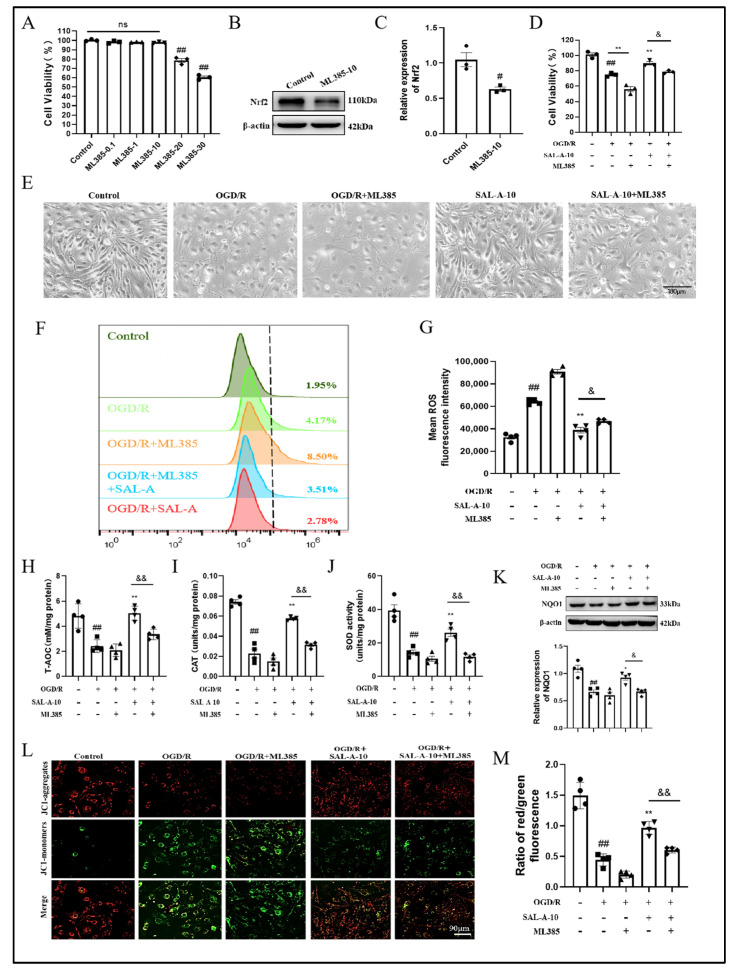
Salvianolic acid A attenuates OGD/R-induced oxidative stress via the Nrf2 signaling pathway in b.End.3 cells. (**A**) Effect of different concentration of ML385 on cell viability (*n* = 3). Representative images of Western blot (**B**) and statistical analysis showing the Nrf2 (**C**) expression in b.End.3 cells after OGD/R (*n* = 3). The cell viability (**D**) and representative images of cell morphology (**E**) in b.End.3 cells after OGD/R (*n* = 3). Representative images (**F**) and quantitative analysis (**G**) of DCFH-DA staining in b.End.3 cells after OGD/R by flow cytometry (*n* = 4). The levels of T-AOC (**H**), CAT (**I**), SOD (**J**) and NQO1 (**K**) in b.End.3 cells (*n* = 4). Representative images of JC-1 staining (**L**) and quantitative analysis of staining (**M**) in b.End.3 cells after OGD/R. Values are expressed as Mean ± SEM. ^#^ *p* < 0.05, ^##^ *p* < 0.01 vs. Control group. * *p* < 0.05, ** *p* < 0.01 vs. OGD/R group. ^&^ *p* < 0.05, ^&&^ *p* < 0.01 vs. OGD/R+SAL-A-10 group.

Nrf2’s effect on SAL-A’s antioxidative stress responses was validated in b.End.3 cells after OGD/R injury. ML385 is a specific inhibitor of Nrf2. Treatment with ML385 at 20 μM and above decreased cell viability (Figure 4A). ML385 10 μM significantly inhibited Nrf2 level and reduced the defensive influence of SAL-A against cell viability decline caused by OGD/R (Figure 4B–D). Cells exhibited morphological changes such as cell shrinkage and dispersion, indicating damage due to oxygen and glucose deprivation after OGD/R. Microscope pictures showed SAL-A could maintain normal cell morphology, but this effect was diminished when ML385 was administered (Figure 4E). DCFH-DA staining is usually used to measure ROS levels. ML385 weakened the inhibitory effect of SAL-A on ROS generation induced by OGD/R, which was confirmed by flow cytometry analysis (Figure 4F,G). Additionally, ML385 hindered the ability of SAL-A to enhance T-AOC, CAT and SOD activities, and increase NQO1 levels (Figure 4H–K). JC-1 staining was used to indicate MMP, with a shift from red to green fluorescence indicating a decline in MMP. SAL-A significantly attenuated the decrease in MMP after OGD/R, while ML385 reduced the protective effect of SAL-A on MMP (Figure 4L,M).

### 2.5. Salvianolic Acid A Attenuates OGD/R-Induced Ferroptosis via the Nrf2 Signaling Pathway in b.End.3 Cells

**Figure 5:**
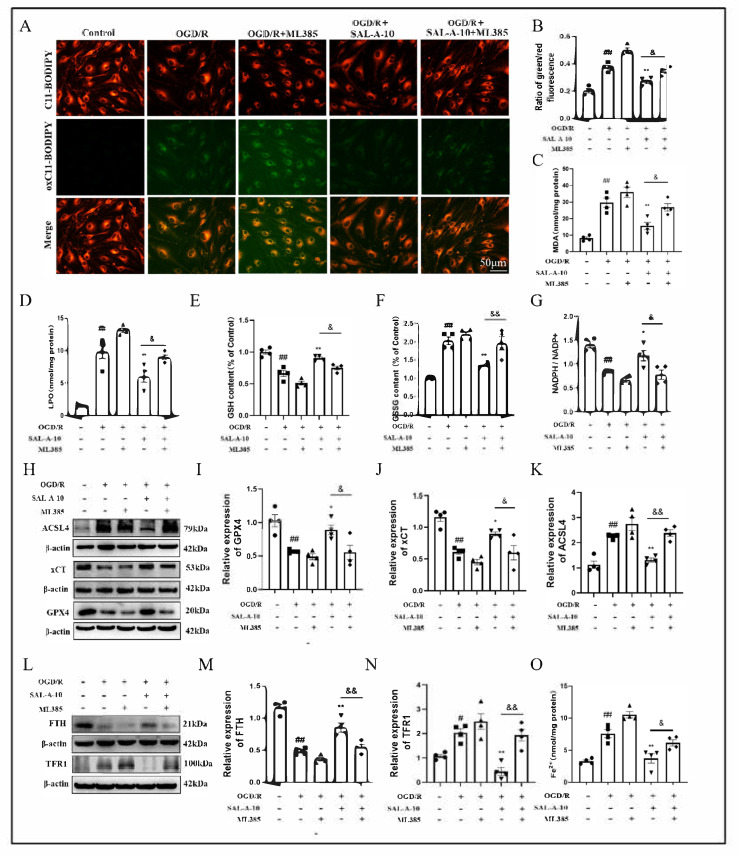
Salvianolic acid A attenuates OGD/R-induced ferroptosis via the Nrf2 signaling pathway in b.End.3 cells. Representative images of BODIPY 581/591 C11 staining (**A**) and quantitative analysis of staining (**B**) in b.End.3 cells after OGD/R. The levels of MDA (**C**), LPO (**D**), GSH (**E**), GSSG (**F**) and NADPH/NADP^+^ (**G**) in b.End.3 cells after OGD/R. Representative images of Western blot (**H**) and statistical analysis showing the ACSL4 (**I**), xCT (**J**) and GPX4 (**K**) expression in b.End.3 cells after OGD/R. Representative images of Western blot (**L**) and statistical analysis showing the FTH (**M**) and TFR1 (**N**) expression in b.End.3 cells after OGD/R. (**O**) The levels of Fe^2+^ in b.End.3 cells after OGD/R. Values are expressed as Mean ± SEM (*n* = 4). ^#^ *p* < 0.05, ^##^ *p* < 0.01 vs. Control group. * *p* < 0.05, ** *p* < 0.01 vs. OGD/R group. ^&^ *p* < 0.05, ^&&^ *p* < 0.01 vs. OGD/R+SAL-A-10 group.

To investigate the role of Nrf2 in the process of SAL-A against ferroptosis, this study conducted ML385 in b.End.3 cells after OGD/R. Firstly, evaluation of lipid peroxidation rates was conducted utilizing BODIPY C11, a probe designed for lipid peroxidation. A shift from red to green fluorescence indicates an increase in lipid peroxidation. The results showed that SAL-A effectively inhibited OGD/R-induced lipid peroxidation, which was attenuated by ML385 (Figure 5A,B). Furthermore, regardless of SAL-A treatment, ML385 increased MDA and LPO levels in cells after OGD/R (Figure 5C,D). Additionally, ML385 inhibited the effects of SAL-A on increasing GSH, NADPH and decreasing GSSG levels after OGD/R (Figure 5E–G). Secondly, the expression of ferroptosis-related proteins was examined. The results indicated that ML385 inhibited the effects of SAL-A in reducing ACSL4 and increasing xCT and GPX4 levels (Figure 5H–K). The effects of SAL-A on decreasing TFR1 and increasing FTH levels after OGD/R were also attenuated by ML385 (Figure 5L–N). Furthermore, ML385 weakened the ability of SAL-A to reduce Fe^2+^ levels after OGD/R (Figure 5O).

## 3. Discussion

Ischemic stroke (IS) is an acute cerebrovascular disease that causes severe damage to brain tissue and CNS function in patients [24]. It is mainly characterized by the occlusion of blood vessels and is intimately linked with arterial atherosclerosis [25]. Diseases such as hypertension or hyperlipidemia lead to altered chemical signals and hemodynamics, which can cause mechanical damage to endothelial cells with chronic vascular inflammation, ultimately contributing to thrombosis [26,27]. The process of PTS depends on photosensitizing dyes, which produce an abundance of oxygen free radicals, when exposed to light of a specific wavelength. These radicals target endothelial cells, causing damage and platelet aggregation, which leads to the development of thrombi [28]. Therefore, PTS effectively simulates the process of thrombus formation in the human body and is suitable for investigating oxidative stress-related injuries. Since thrombosis often begins with damage to the vascular endothelium and ferroptosis is closely related to lipid peroxidation caused by free radical damage [29,30], the PTS model was chosen for this study and the inhibitory effect of SAL-A on ferroptosis was investigated.

SAL-A is a phenolic acid natural product with multiple pharmacological activities. It has been demonstrated to exert therapeutic effects on IS through various signaling pathways. For instance, SAL-A inhibits cell apoptosis induced by IS through protein kinase A (PKA) and improves cognitive impairment [31]. SAL-A improves chronic cerebral ischemic injury by inhibiting the activation of nuclear factor kappa-B (NF-κB) via dopamine receptors D2 (Drd2) [32]. Additionally, some studies have indicated that SAL-A regulates the metabolic disturbances in the brain induced by IS, with effects that are more pronounced at the brain tissue level than in the serum. This suggests that due to the disruption of the blood-brain barrier, the concentration of SAL-A in the central nervous system increases, allowing it to exert its effects primarily within the brain [33]. Although the effects of the PTS model have been well studied and used in relevant drug studies, there is still a lack of evidence for a protective effect of SAL-A in PTS mice. Our results demonstrate that SAL-A improves neurobehavioral deficits and reduces the infarct area in PTS mice, with the best therapeutic effect observed at 10 mg/kg SAL-A. Additionally, the HE staining result indicate that SAL-A alleviates brain cell and neuronal damage in PTS mice.

The relationship between oxidative stress and ferroptosis is close, as both are based on electron transfer reactions and can be induced through shared molecular pathways [34]. Recent research shows that inhibitors targeting ferroptosis can alleviate the harm inflicted by oxidative stress on nerve cells [35]. Our results indicate that SAL-A enhances the brain’s antioxidant capacity by increasing the activities of CAT and SOD in PTS mice. NADPH has been shown to have neuroprotective effects in IS, primarily through its involvement in antioxidant stress reduction and improvement of energy metabolism [36]. In this study, SAL-A can elevate NADPH levels and suppress the expression of NOX2, SOD and NOX4, which induce ROS generation, thereby alleviating oxidative stress damage. Nrf2 plays a critical endogenous role in the response to antioxidant stress. It has been observed that various compounds, such as caffeic acid, can activate Nrf2 and downstream proteins, causing the diminution of oxidative stress and diminishing the area of infarction and brain harm [37]. This study demonstrated that SAL-A enhances Nrf2 expression and downstream antioxidant enzyme expression in the brains of PTS mice. Ferroptosis plays a significant role in IS, including two features: lipid and iron metabolism disorder [38]. The role of SAL-A against ferroptosis in IS was clarified for the first time in this study. ACSL4 catalyzes the acylation of long-chain polyunsaturated fatty acids to produce lipid peroxides that undergo esterification via interaction with membrane phospholipids and then boost ferroptosis [39]. The results in this study indicate that SAL-A can reduce the levels of MDA and the expression of lipid oxidation-related enzyme ACSL4 in PTS mice, indicating inhibition of lipid peroxidation processes. GSH serves to remove peroxides, and depletion of GSH renders cells susceptible to ferroptosis [40]. Our results demonstrate that SAL-A can increase GSH levels and enhance GSH’s reducing capacity by upregulating the expression of GPX4 and xCT. Iron levels are crucial for inducing ferroptosis, and SAL-A can reduce intracellular iron content and restrict iron transport by inhibiting TFR1.

Nrf2 is pivotal in fighting oxidative harm, including lipid peroxidation, and is closely associated with ferroptosis [41,42]. The latest research indicates that lipid peroxidation may trigger the Nrf2 pathway in cases of hepatocellular carcinoma [43]. Two known inducers of ferroptosis, RSL-3 and Erastin, initiate the ferroptosis cascade by inhibiting both the antioxidant capacity of GSH and its synthesis, which are also targets of Nrf2 [44]. As observed through Nrf2 inhibition experiments, the defensive role of SAL-A against damage caused by OGD/R in cells was reduced when using the inhibitor ML385. Excessive production of ROS is an important indicator of oxidative stress [45]. The ability of SAL-A to inhibit ROS generation, enhance antioxidant levels, and protect mitochondrial membrane potential is weakened by ML385, indicating an obstruction of SAL-A’s antioxidative stress effects. Moreover, inhibition of Nrf2 further exacerbated OGD/R-induced ferroptosis in the presence of SAL-A. The ability of SAL-A to reduce lipid peroxides and increase GSH levels was diminished when Nrf2 was inhibited, and the levels of ACSL4 was enhanced, and GPX4 and xCT were suppressed. Additionally, it has been shown that Nrf2 is vital in several metabolic processes, such as iron and heme metabolism. Therefore, targeting Nrf2 remains a highly feasible approach in diseases characterized by lipid peroxidation and ferroptosis, particularly IS. The iron-related cascade of ferroptosis was reactivated, as SAL-A’s ability to reduce iron levels was impeded, and its ability to inhibit TFR expression was hindered. Additionally, the levels of ferritin-related protein FTH decreased when Nrf2 was inhibited.

Based on our findings, we propose that SAL-A regulates the process of ferroptosis by enhancing the expression of Nrf2, which affects its downstream target proteins that mediate the inhibition of ferroptosis. The increased expression of Nrf2 has beneficial effects, including the elevation of GPX4 and xCT levels, as well as the reduction of ACSL4, which together help counteract lipid peroxidation and decrease the levels of lipid peroxidation markers such as ROS, MDA, and LPO. Additionally, SAL-A can suppress iron intake by lowering TFR1 levels and increase FTH levels to reduce intracellular free iron, thereby regulating intracellular iron levels. The protective effect of SAL-A against ferroptosis was significantly attenuated when Nrf2 was inhibited, highlighting the crucial role of Nrf2 in SAL-A’s anti-ferroptosis effects.

There are still some limitations in our study. For the effects of SAL-A on Nrf2 expression, we observed only an upregulation effect of Nrf2 by SAL-A, which was diminished in the presence of ML385. However, since ML385 may have more targets than just Nrf2, it remains unclear how SAL-A promotes Nrf2 and how it alters Nrf2 activity. This requires further investigation. In our upcoming experiments, we will examine whether the Nrf2 knockout model can mitigate the effects of SAL-A and examine changes in GPX4 expression or activity following treatment with the ferroptosis-specific inhibitor ferrostatin-1 to explore the specific mechanism by which SAL-A regulates Nrf2.

## 4. Materials and Methods

### 4.1. Reagents

SAL-A (purity > 98%) was provided by Prof. Yang Lv from the Institute of Materia Medica (Beijing, China). For cell culture, Dulbecco’s modified Eagle’s medium (DMEM) was purchased from Procell Life Science&Technology (PM150210, WuHan, China). Fetal bovine serum (FBS) was purchased from PAN (P30-3306, Adenbach, German); 2, 3, 5-triphenyltetrazolium chloride (TTC) was purchased from Sigma-Aldrich (T8877, Darmstadt, Germany); TUNEL cell apoptosis detection kit (G1504-50T) was purchased from Servicebio Technology (Wuhan, China); Activity assay kits of total antioxidant capacity (T-AOC, E2016), superoxide dismutase (SOD, E2011), catalase (CAT, E2030) were purchased from Applygen (Beijing, China). Content assay kits of lipid peroxidation (LPO, BC5245), malondialdehyde (MDA, BC5245), GSH (BC5245), GSH oxidized (GSSG, BC5245), NADPH (BC1105), iron (Fe, BC4355), ferrous iron (Fe^2+^, BC5415) were purchased from Solarbio Biotechnology (Beijing, China); Mitochondrial membrane potential (MMP) assay kit with JC-1 (C2003S) and ROS assay kit (DCFH-DA) were purchased from Beyotime Biotechnology (Shanghai, China); BODIPY C11 581/591 probe was purchased from Thermo Fisher Scientific Inc. (Waltham, MA, USA).

### 4.2. PTS Mice Model and SAL-A Treatment

C57BL/6J mice (SPF-grade, 20–24 g, Male, Quality certificate number: SCXK (Beijing) 2021-0006) were purchased from Vital River Laboratory Animal Technology Co., Ltd. (Beijing, China). Mouse manipulation was licensed (Certificate number.: 00008688) by the Ethics Committee of Peking Union Medical College. The survival environment of C57BL/6J mice was parameterized (25 °C, 50% humidity) and a 12 h photoperiod was simulated. Mice were randomly assigned to 5 groups after the model building: (1) Sham group: underwent the sham operation and was orally administered saline (0.1 mL/10 g) for 7 days; (2) Model group: underwent the PTS operation and was orally administered saline (0.1 mL/10 g) on the same day and continued for 7 days; (3) SAL-A group-1 mg/kg (SAL-A-1): after the PTS, mice were orally administered 1 mg/kg SAL-A (0.1 mL/10 g) for 7 days; (4) SAL-A group-3 mg/kg (SAL-A-3): after the PTS, mice were orally administered 3 mg/kg SAL-A (0.1 mL/10 g) for 7 days; (5) SAL-A group-10 mg/kg (SAL-A-10): after the PTS, mice were orally administered 10 mg/kg SAL-A (0.1 mL/10 g) for 7 days. The SAL-A group received the first dose 1.5 h after the PTS, followed by daily administrations at the same time for a total of 7 consecutive days. Both the Sham and the Model group were administered saline 1.5 h after the PTS, followed by daily saline treatment at the same time for 7 consecutive days. Each mouse’s body weight was consistently recorded every day before the SAL-A and saline administration (once daily).

Briefly, after acclimatized for 3 days, rats were anesthetized by 3% isoflurane for induction and 2% isoflurane-maintained anesthesia. A tail vein injection of 10 mg/mL Rose Bengal (0.1 mL/10 g) was given to mice. Mouse head was fixed using a stereotaxic instrument, with body at condition of 37 °C. The midline of the mouse’s head was incised, exposing the bregma point. The coordinates of the bregma point (X, Y) were recorded. A laser device (wavelength 532 nm, power 100 mW, spot diameter 2 mm) was used to irradiate a specific position (X + 0.1 cm, Y + 0.1 cm) for 30 min. After the irradiation, surface disinfection of the skin was performed using iodophor. The sham group experienced an identical process, differing in the use of saline in place of Rose Bengal.

### 4.3. Behavior Tests

#### 4.3.1. Neurological Deficit Scoring (mNSS) and Zea Longa Scoring

Neurological impairments were evaluated using mNSS, an accepted scoring benchmark for neurological impairments outcomes (normal = 0; severe nerve damage = 18), on the 7 day following the PTS [46]. The Zea Longa assessment comprises: 0 points, indicating no behavioral issues; 1 point, signifying the incapacity to stretch the forelimb across the injury; 2 points, indicating a circular motion towards the hemiplegic side while walking; 3 points, indicating an inclination towards the side with hemiplegia during walking; 4 points, signifying an inability to walk [47]. As the scores in mice rose, so did the intensity of neurological harm [48].

#### 4.3.2. Corner Test

The corner test was conducted as in previous studies [49]. The test device consists of two transparent panels made of acrylic, with an angle of 30 degrees between the panels. Before executing corner tests on each mouse, the testing apparatus was purified using 75% ethyl alcohol. The mice were acclimatized for 10 min when first placed, and the direction of their entire turn was documented. A total of 20 recordings were made at 10 min intervals for each mouse.

#### 4.3.3. Grip Strength Test

The mice are placed on the grip strength gauge (BIO-GS4, Columbus Instruments, Columbus, OH, USA). Setting the parameter to record the maximum tensile force. Subsequently, every mouse was dragged back at a steady pace with its tail until the tension shown ceased to rise [50]. Ten recordings were made at 10 min intervals for each mouse. The average of the 10 trials was taken as the average grip strength of each mouse.

### 4.4. Infarct Volume Assessments

Brains of the PTS mice were obtained on day 7. Euthanasia of mice by exposure to an environment containing carbon dioxide (40%) for 10 min. The brains were maintained at −20 °C for 35 min and sectioned into 6 parts (2 mm each). The slices were placed in a thermostat at 37 °C for 45 min at 2% TTC. Then, 4% paraformaldehyde (PFA) was used to soak the slices for 12 h and recorded by a high-definition camera. Infarct areas were measured using ImageJ software (version 1.8.0.372). Infarct volume = infarct area / total area × 100% [51].

### 4.5. Hematoxylin-Eosin (HE) Staining

On day 7 after the PTS assay, the mice were maintained at deep anesthesia and perfused with 4% PFA through their left ventricles, allowing 4% PFA to flow out of the right apices. Coronal slices underwent fixation in 4% PFA for a duration of 36 h, followed by encasement in paraffin. Then, deparaffinized in xylene and immersed in alcohol to induce brain dehydration and sectioned into 3.5 μm sections. Sectors prepared in paraffin underwent a 5 min treatment with hematoxylin followed by 2 min eosin treatment. At long last, the optical microscope revealed alterations in brain tissues [52].

### 4.6. Transmission Electron Microscopy (TEM) Assay of Mitochondrial Morphology

On the 7th day after the PTS assay, the mice were maintained at deep anesthesia and a region at the edge of the brain infarction was dissected. The tissue was transferred to the fixative solution for electron microscopy for 2 h. Osmium tetroxide solution (1%) was used to preserve tissue samples for 1 h, followed by dehydration. Then, the tissue was embedded at 37 °C overnight. Subsequently, the ultra-thin sections (70 nm) of tissue were immersed in 2% uranyl acetate solution and 2.6% lead citrate solution for 8 min. The thoroughly dried samples were analyzed after washing in distilled water by TEM (UC7, Leica biosystems, Wetzlar, Germany) [53].

### 4.7. DAB Enhanced Prussian Blue Stain

Following the instructions, paraffin sections were stained with Prussian blue A and B staining solution in equal volume at room temperature for 1 h. After removing the staining solution, Prussian blue staining solution C is added to the tissue for 5 min. Then, DAB Staining Solution is added to the tissue for 30 min. After the staining solution is removed, the slices are conducted with HE staining and observed under the microscope [54].

### 4.8. Content Assay of T-AOC, CAT, SOD, GSH, GSSG, NADPH, Fe and Fe^2+^

Information about kits associated with these indicators is available above. The aforementioned kits were executed following the guidelines issued by the kit producers and served to measure the concentrations of the aforementioned markers in the b.End.3 cells and cerebral tissue of PTS mice.

### 4.9. Oxygen-Glucose Deprivation/Reoxygenation (OGD/R) Modeling and Grouping

Brain microvascular endothelial (b.End.3) cells were purchased from Institute of Basic Medical Sciences (Beijing, China). DMEM mixed with 10% FBS was used for cell culture in a suitable environment (37 °C, 5% CO_2_). To produce cerebral ischemic injury model, OGD/R was exerted. The cells were transferred to a low-oxygen incubator (O_2_ = 1%, Thermo Fisher Scientific, USA) and maintained in No Glucose-DMEM (PM150270, Procell Life) for 12 h. After OGD, the cell culture was transferred to the suitable environment mentioned above for 8 h. b.End.3 cells were categorized into five groups: (1) Control group: Cells were grown in suitable environment. (2) OGD/R group: Cells underwent OGD/R damage. (3) OGD/R+ML385 group: After administered with ML385 for 2 h, cells underwent OGD/R damage. (4) SAL-A-10 group: Cells underwent OGD/R damage and administered with SAL-A (10 μM) in reoxygenation. (5) SAL-A-10+ML385 group: After administered with ML385 for 2 h, cells underwent OGD/R damage and administered with SAL-A (10 μM) in reoxygenation. For all cells in the SAL-A treatment group, SAL-A was administered immediately after the OGD/R [55].

### 4.10. Cell Viability Assay with CCK-8

b.End.3 cells were seeded in a 96-well plate (5 × 10^3^ cells/well). After OGD/R and drug treatment was performed, the supernatant was removed. Every well received an addition of 10 μL of CCK-8 reagent. The plate was maintained at 37 °C for a duration of 2 h and was protected from light. OD measured at 450 nm.

### 4.11. ROS Analysis

After the supernatant was removed, cells were subjected to 2,7-dichlorodi-hydrofluorescein diacetate (DCFH-DA) for 45 min. After removing the dye solution and adding PBS, observations and analyses of cells were conducted using flow cytometry provided by Becton, Dickinson company. During incubation, intracellular ROS react with DCFH-DA and emit green fluorescence. The microplate reader (2104, Multilabel Reader, PerkinElmer, USA) was utilized to ascertain the mean fluorescence intensity (MFI) of the ROS level (% of Control) [56].

### 4.12. MMP Analysis with JC-1 Staining

The MMP assay kit was used in accordance with guidelines issued by the kit producers to examine the changes in MMP induced by OGD/R. JC-1 staining dye was co-incubated with cells in 96-well plates for 45 min. Then, the plates were shielded against light for 35 min. After removing the dye solution and adding PBS before the samples were observed using a fluorescence microscope. If the MMP of the cells is high, they will show red fluorescence, and they will show green fluorescence in the opposite case. The microplate reader was utilized to ascertain the red-green fluorescence ratio [57].

### 4.13. Lipid Peroxidation Analysis with BODIPY 581/581 C11 Staining

The application of BODIPY 581/591 C11 staining is frequently utilized for identifying lipid peroxidation within cells. When lipid peroxidation occurs, it results in green fluorescence. Cells in each group are subjected to BODIPY 581/581 C11 probe diluted by PBS (2 μM) for 20 min. Then, observed by fluorescence microscope. The red-green fluorescence ratio of each well was detected by microplate reader [58].

### 4.14. Western Blotting Analysis

Euthanasia of mice by exposure to an environment containing carbon dioxide (40%) for 10 min. Then, brain tissues were removed and kept in frozen condition for further analysis. Proteins were isolated using RIPA lysates containing phosphatase inhibitors (P1260-1, Applygen) and protease inhibitors (P1265-1, Applygen). Protein stock solution was mixed with loading buffer (B1007-5, Applygen) at 100 °C for 10 min to prepare electrophoretic protein samples. Protein samples underwent separation through electrophoresis (80 V, 80 mA) by sodium dodecyl sulfate-polyacrylamide gel (SDS-PAGE). Subsequently, the protein was loaded using a polyvinylidene fluoride (PVDF) membrane. The membranes underwent horizontal slicing based on molecular size indicators and were then incubated with antibodies for 16 h. Following the elimination of primary antibodies, the membranes underwent a 2 h incubation at 25 °C with horseradish peroxidase-linked secondary antibodies. Finally, membranes were immersed by ECL luminescent solution (W0001, Applygen) and measured using gel imaging system (5200, Tanon, Beijing, China). The intensity of the bands was analyzed using ImageJ software and each band used the intensity of anti-β-actin as a loading control for normalized. If necessary, PVDF membranes are stripped and the above steps are repeated [59].

### 4.15. Statistical Analysis

GraphPad Prism software version 7.0 (version 9.3.1) was used to compare the differences between each group. Data were presented as mean ± SEM and from at least three independent experiments performed in duplicate or triplicate. One-way ANOVA was used to calculate differences between the various groups. Tukey’s multiple comparison post hoc test was performed to determine significance levels. Statistical significance was considered at *p* < 0.05.

## 5. Conclusions

In conclusion, SAL-A activates Nrf2 and regulates its downstream key factors, including essential players in lipid peroxidation resistance, such as GPX4, xCT, and ACSL4, leading to a reduction in the production of MDA and ROS. Furthermore, by modulating the levels of FTH and TFR1, SAL-A decreases intracellular free iron, ultimately inhibiting ferroptosis. These findings provide compelling experimental evidence and a molecular basis for the therapeutic potential of SAL-A in treating ischemic stroke and mitigating ischemic brain injury.

## Data Availability

The data of this study are available from the corresponding author upon reasonable request.

## Abbreviations

AREs	antioxidant response elements
ACSL4	long-chain acyl-CoA synthetase 4
b.End.3	brain microvascular endothelial cells
CAT	catalase
Dr2	dopamine receptors D2
DCFH-DA	2,7-dichlorodi-hydrofluorescein diacetate
FTH	ferritin heavy chain
GPX4	glutathione peroxidase 4
GSH	glutathione
GSSG	oxidized glutathione
HO-1	heme oxygenase 1
HE	Hematoxylin-Eosin
IS	Ischemic stroke
Keap1	kelch-like ech-associated protein 1
LPO	lipid peroxidation
MDA	malondialdehyde
MMP	mitochondrial membrane potential
mNSS	neurological deficit scoring
NADPH	reduced form of nicotinamide adenine dinucleotide phosphate
NF-κB	nuclear factor kappa-B
NQO1	NADPH quinone oxidoreductase 1
Nrf2	nuclear factor erythroid 2-related factor 2
NOX	NADPH oxidases
OGD/R	oxygen-glucose deprivation/reoxygenation
PTS	photochemical induction of stroke
PPARγ	peroxisome proliferators-activated receptors γ
PUFAs	polyunsaturated fatty acids
PKA	protein kinase A
ROS	reactive oxygen species
SAL-A	salvianolic acid A
SLC7A11/xCT	solute carrier family 7 member 11
SOD	superoxide dismutase
TFR1	transferrin receptor 1
TTC	2, 3, 5-triphenyltetrazolium chloride
T-AOC	total antioxidant capacity
TEM	transmission electron microscopy
